# Genome Scale Modeling to Study the Metabolic Competition between Cells in the Tumor Microenvironment

**DOI:** 10.3390/cancers13184609

**Published:** 2021-09-14

**Authors:** Itziar Frades, Carles Foguet, Marta Cascante, Marcos J. Araúzo-Bravo

**Affiliations:** 1 Computational Biology and Systems Biomedicine Group, Biodonostia Health Research Institute, 20009 San Sebastian, Spain; itziar.frades@biodonostia.org; 2 Department of Biochemistry and Molecular Biomedicine, Institute of Biomedicine of University of Barcelona, Faculty of Biology, Universitat de Barcelona, Av. Diagonal 643, 08028 Barcelona, Spain; cfoguet@ub.edu (C.F.); martacascante@ub.edu (M.C.); 3 Centro de Investigación Biomédica en Red de Enfermedades Hepáticas y Digestivas (CIBEREHD) (CB17/04/00023) and Metabolomics Node at Spanish National Bioinformatics Institute (INB-ISCIII-ES-ELIXIR), Instituto de Salud Carlos III (ISCIII), 28020 Madrid, Spain; 4 Max Planck Institute of Molecular Biomedicine, 48167 Münster, Germany; 5 Centro de Investigación Biomédica en Red de Fragilidad y Envejecimiento Saludable (CIBERfes), 28015 Madrid, Spain; 6 Translational Bioinformatics Network (TransBioNet), 8001 Barcelona, Spain; 7 Ikerbasque, Basque Foundation for Science, 48012 Bilbao, Spain

**Keywords:** metabolic reprogramming in cancer, immune system, genome-scale metabolic models, constraint-based modeling, stoichiometric models, kinetic metabolic models, metabolic crosstalk

## Abstract

**Simple Summary:**

Immune and cancer cells compete for nutrients within the tumor microenvironment, leading to a metabolic battle between these cell populations. In this battle, tumor cells reprogram their metabolism for a high demand of building blocks and energy and to gain advantages over immune cells. To study these mechanisms, we require the quantification of metabolic fluxes, which can be estimated at the genome-scale, with constraint-based or kinetic modeling.

**Abstract:**

The tumor’s physiology emerges from the dynamic interplay of numerous cell types, such as cancer cells, immune cells and stromal cells, within the tumor microenvironment. Immune and cancer cells compete for nutrients within the tumor microenvironment, leading to a metabolic battle between these cell populations. Tumor cells can reprogram their metabolism to meet the high demand of building blocks and ATP for proliferation, and to gain an advantage over the action of immune cells. The study of the metabolic reprogramming mechanisms underlying cancer requires the quantification of metabolic fluxes which can be estimated at the genome-scale with constraint-based or kinetic modeling. Constraint-based models use a set of linear constraints to simulate steady-state metabolic fluxes, whereas kinetic models can simulate both the transient behavior and steady-state values of cellular fluxes and concentrations. The integration of cell- or tissue-specific data enables the construction of context-specific models that reflect cell-type- or tissue-specific metabolic properties. While the available modeling frameworks enable limited modeling of the metabolic crosstalk between tumor and immune cells in the tumor stroma, future developments will likely involve new hybrid kinetic/stoichiometric formulations.

## 1. Introduction

**Figure 1:**
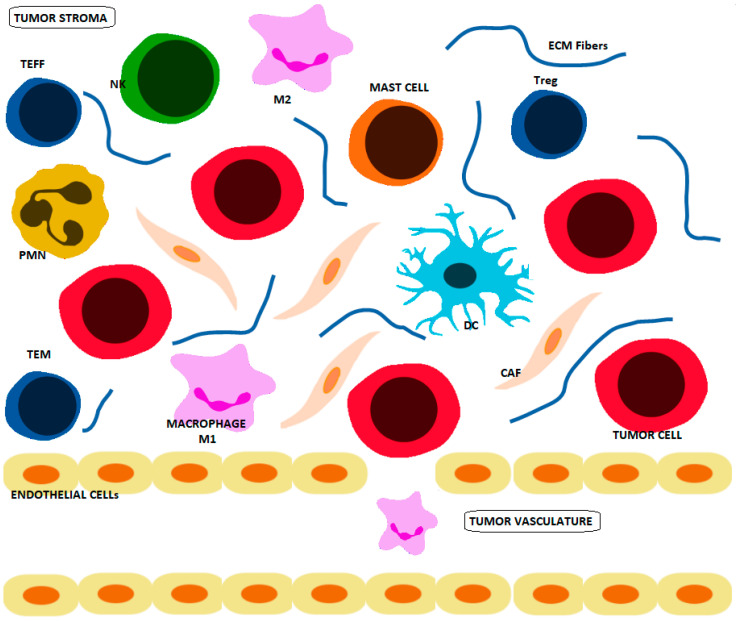
The cell types within the TME include cancer cells, immune cells and stromal cells. The immune cells in the TME include natural killer (NK) cells, dendritic cells (DC), macrophages (M1 and M2), polymorphonuclear leukocytes (PMN) (including neutrophils, eosinophils and basophils), mast cells and T cells (Regulatory T cells (Tregs), effector T cells (TEFF) and memory T cells (TMEM)). The stromal cells include cancer-associated fibroblasts (CAFs), which are embedded in the TME with Extracellular Matrix (ECM) fibers.

Tumors have a pseudo-organ-like dynamism with numerous cell types that interact to create a unique physiology [1]. The cell types within the tumor microenvironment (TME) include cancer cells, immune cells and stromal cells (Figure 1), and they play a key role in the progression of the disease [2]. Immune response can potentially lead to the destruction of cancer cells; however, tumors often evade the host’s immune system through different mechanisms [3]. Indeed, the dynamic interplay between tumor, stroma and immune system can lead to both pro-tumorigenic and anti-tumorigenic behaviors [4,5]. At the metabolic level, there is a battle between immune and cancer cells where they compete for nutrients within the TME. Cancer cells reprogram their metabolism to have an increased nutrient uptake from the TME, which limits the availability of nutrients for immune cell populations, weakening, corrupting and evading immunosurveillance [6].

The immune effectors against cancer are natural killer cells (NK), dendritic cells (DC), macrophages, polymorphonuclear leukocytes (PMN) (neutrophils, eosinophils and basophils), mast cells and T cells (lymphocytes B and cytotoxic T lymphocytes). NK cells, DC, PMN, mast cells and macrophages constitute the first-line effectors to cancer cells (Figure 1). NK cells participate in the innate and adaptive immune response through close interactions with T lymphocytes with cytotoxic effects and memory [6].

Based on the expression of cell surface markers T cells are classified into CD4+ helper T (TH) and CD8+ cytotoxic T (TC) functional cell lineages. TH cells bind to the class II major histocompatibility complex (MHC) molecules and help the immune system recognize foreign substances to modulate the immune response and to produce cytokines to enhance or suppress inflammation [7]. TC cells target pathogen-infected or malignant cells by secreting inflammatory cytokines, as well as cytotoxic proteins that act as cell lytic molecules, such as the pore-forming protein perforin, which facilitates the entry of the inducing apoptosis granzyme in the target-cell membrane. TH cells circulate through the body, and they are activated when recognize an antigen presented by the antigen-presenting cell (APC). Antigen-presenting starts with the ingestion of the foreign particles by the APC; then the particles are degraded and exported to the cell surface, with the resulting antigens, where they are presented in association with molecules from the class II MHC. Upon activation, TH cell populations expand and release several immunomodulating cytokines [8]. TC cells travel through the blood flow, searching for antigens that are presented by MHC Class I to the T-Cell Receptor (TCR). The activation of these cells required several interactions between the molecules they express on the surface, the molecules on the surface of the APCs and DCs [9].

The TC activation requires two signals: a first signal the TCR binds to the MHC class I molecule, and a second signal assisted by stimulating the CD28 molecule on the TC with cytokines released from TH cells. Then, using IL-2 acting as a growth and differentiation factor for the TC, the TC clonally expand, increasing the cells that recognize the antigen [10].

Before encountering an antigen, T cells are called T naïve (TN) and after antigen encounter, T cells are activated. Regulatory T cells (Tregs) emerge from the acquisition of immuno-suppressive characteristics of the TH cells. TC cells can differentiate from TN to stem central memory T cells (TSCM), to central memory T cells (TCM), effector memory T (TEM) or effector T cells (TEFF). All of these cells finally converge in terminally differentiated effector memory cells re-expressing CD45RA (TEMRA) [7]. TN cells usually express CD45RA cell-surface antigen isoform when activated; T cells, such as TCM and TEM cells, switch from expressing isoform CD45RA to the isoform CD45RO [11,12], whereas TEMRAs, such as TN cells, re-express CD45RA [13].

Memory T cells can be CD4+ or CD8+ and are TEM or TCM subtype depending on the set of cell surface markers they express. All of them are long-lived and capable of expanding quickly when re-exposed to their corresponding antigen, which provides a controlled immune response against previously encountered foreign bodies.

**Figure 2:**
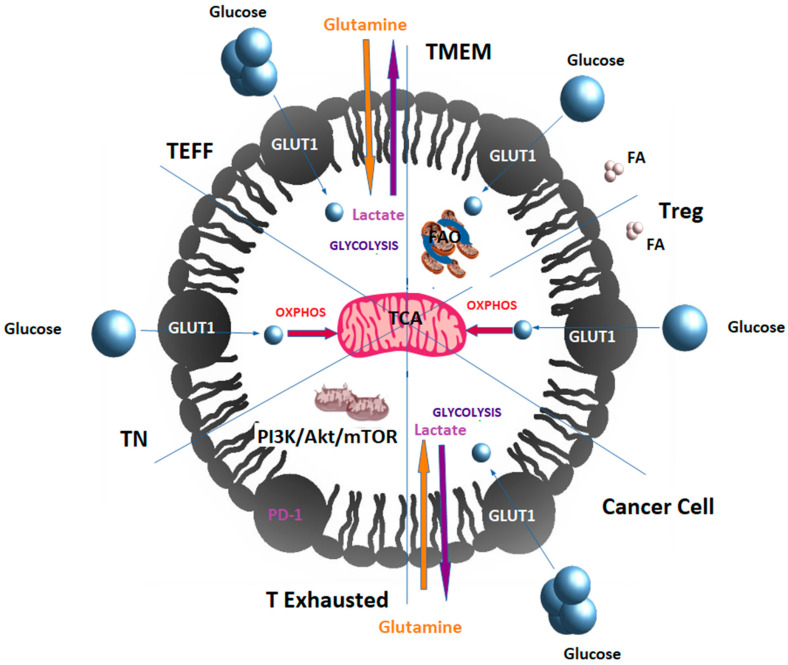
T cells remodel their metabolism to get differentiated into T-cell subsets within the TME. TN cells use glucose that enters in OXPHOS. Upon encountering cognate antigen, activated T cells get differentiated into TEFF, rapidly uptaking glucose and glutamine to perform aerobic glycolysis and generating lactate as a by-product. Once the antigen is cleared, TEFFs can get differentiated into memory T cells (TMEM), which depend on fatty acid oxidation. Equally, cancer cells depend on aerobic glycolysis and glutaminolysis that are fueled by glucose and glutamine, respectively, with production of lactate as a by-product. TEFF within the TME can be differentiated into Tregs with immunosuppressive properties that mainly produce energy by oxidative phosphorylation (OXPHOS) and fatty acid oxidation (FAO). T cells can become exhausted if they fail to clear antigens, as, for example, in cancer. T lymphocytes derived from tumors show elevated levels of PD-1 decreasing PI3K/Akt/mTOR signaling pathway and therefore glycolysis. Exhausted T cells in tumors having often dysfunctional mitochondria rely on FAO. FA: fatty acids. Cancer cells and T cells compete for nutrients, since cancer cells also have an increased glucose and glutamine uptake and they use aerobic glycolysis with production of lactate that is exported to the extracellular space, promoting acidification in the TME that compromises the activity of immune cells.

T cells rewire their metabolism to promote these changes. TN cells primarily rely on glucose respiration and oxidative phosphorylation (OXPHOS) to meet their energy needs. Upon recognizing an antigen, activated T cells differentiate to TEFF and switch to aerobic glycolysis, increasing both glucose and glutamine uptake [14]. Once the antigen is cleared, memory T cells switch to fatty acid oxidation (FAO) as their primary energy source [14]. Similarly, Tregs mainly depend on OXPHOS and FAO [15]. T cells become exhausted when they fail to clear antigens. T lymphocytes derived from tumors show elevated levels of PD-1 (Programmed Cell Death-1), decreasing the PI3K/Akt/mTOR signaling pathway’s activity. PD-1 and PD-L1 (Programmed Death-Ligand 1) belong to the family of immune checkpoint proteins that modulate the T-cell response [16]. The PD-1/PD-L1 interaction ensures that the immune system is activated only at the appropriate time in order to avoid chronic autoimmune inflammation and represents an adaptive immune resistance mechanism of tumor cells in response to endogenous immune antitumor activity [17]. Indeed, tumor cells often overexpress PD-L1, which binds to the activated T cells’ PD-1 receptors and inhibits their cytotoxic activity [18]. Exhausted T cells in tumors with elevated levels of PD-1 are unable to uptake nutrients, such as glucose and glutamine; therefore, even if they often have dysfunctional mitochondria and decreased mitochondrial mass, they rely on FAO [14,19] (Figure 2). It has been suggested that the degree of T-cell exhaustion induced by PD-1 might depend on the reserves of endogenous lipids that can be mobilized for energy generation [20].

The metabolic phenotype of cancer cells reflects the characteristics of any proliferating cell; thus, cancer and immune cells have similar metabolic requirements [21], and the metabolism is responsible for many of the pathophysiologic interactions that occur within the TME, including the symbiotic nutrient sharing, competition for nutrients and usage of metabolites as signaling molecules [1]. A full understanding of the metabolic phenotype and crosstalk between cancer cells and immune cell populations requires tracing metabolic fluxes (i.e., the rate at which metabolites are interconverted through metabolic reactions and transported across cellular membranes). In this regard, over the last decade, Genome-Scale Models (GEMs) have emerged as platforms where multiple layers of data can be integrated to estimate the underlying flux maps at a genome-scale. Thus, in addition to other aims, GEMs can be applied to the study of the interactions of cancer cells with the surrounding cells from the TME [22], as well as predicting [23] and circumventing the drug resistance [24].

### 1.1. Crosstalk between Cancer and Immune Cells

**Figure 3:**
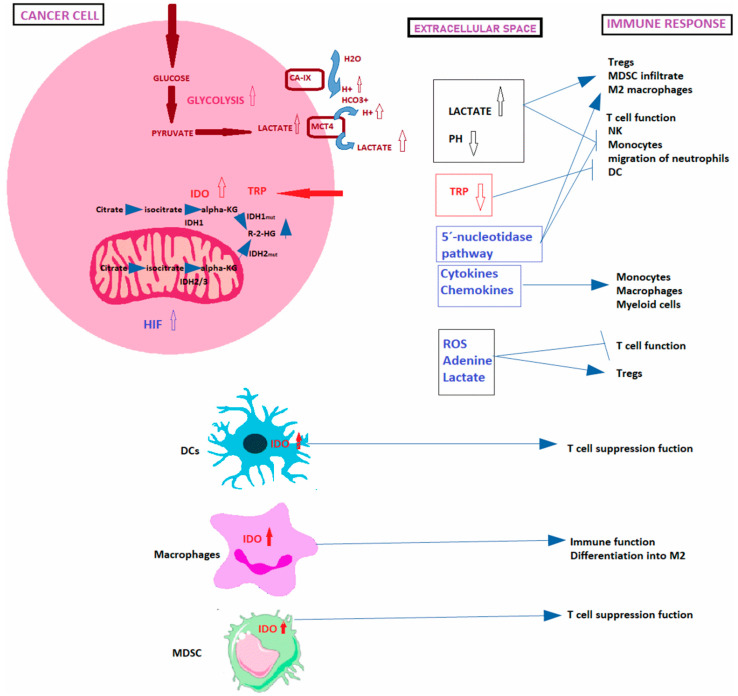
Mechanisms of immunosurveillance scape. Including lactate and proton release by cancer cells within the TME and the consecutive immune response, consequences of indoleamine 2,3 dioxygenase (IDO) overexpression and hypoxia and hypoxia inducible factor (HIF) production by cancer cells on the antitumoral immune response, and lastly Isocitrate dehydrogenase (IDH) mutations in cancer cells, promoting the accumulation of R-2-HG oncometabolite. Dendritic cells (DCs), tryptophan (TRP), myeloid-derived suppressor cells (MDSC); Reactive Oxygen Species (ROS); natural killer cells (NK); dendritic cells (DC), regulatory T cells (Tregs).

Metabolic reprogramming and immune escape are two major hallmarks of cancer cells. Metabolic reprogramming is not only indispensable for sustaining tumorigenesis but also for maintaining homeostasis of immune cells. Immune and tumor cells compete for metabolic resources within the TME, as they share metabolic needs. However, compared with immune cells, cancer cells show greater plasticity to reshape their metabolism to circumvent the adverse conditions and increase the ability to capture and exploit the limited metabolites available within the TME. This leads to an interaction between tumor and immune cells that results in a metabolic competition within TME that limits nutrient disposition and conditions the function of immune cells and often results in immunosuppression [25,26]. Indeed, cancer metabolic reprograming creates a metabolically hostile TME in which immune cells acquire functional defects and enter a hyporesponsive (or anergic) reversible state, with impaired effector capacities even favoring tumor progression [27]. Local nutrient depletion or production of metabolic “waste” products can affect immune cells contributing to immune evasion in several ways (Figure 3) [28]. In this regard, it has been identified critical differences between immune and tumor cells which may be exploited to treat cancer [21]. Thus, the blocking of tumor-mediated immunosuppression, the tailoring of immune responses by manipulating cellular metabolic pathways and the identification of new targets have proven to have a very positive impact in clinical oncology [15,29].

Cancer triggers immune escape thought several mechanisms including reduced expression of antigens at the surface of tumor cells, reduced expression of MHC molecules by APCs, impaired co-localization of TCR and co-stimulatory receptors, secretion of inhibitory cytokines and activation of inhibitory receptors on T-cell surface, as well as metabolic rewiring [30]. The influence of metabolic determinants on the regulation of immune cells plasticity and function are still to be fully elucidated. Emerging evidence suggests that metabolic crosstalk between cancer and immune cells can strongly contribute to immune suppression and can even facilitate cancer progression [6,14,28]. Since the loss of physiological regulation in cancer cells is associated with increased metabolic demand and each type of immune cell has distinct metabolic requirements that restrict or alter cell fate, the interaction of cancer cells and immune cells is subject to a significant plasticity to engage metabolic programs that modulate metabolite levels and consequently their function and fate [21]. Consequently, metabolic pathophysiologic interactions in the TME that drive cancer progression derive from autonomous malignant cells alone or with interacting cells rewiring their metabolic properties. The main metabolic reprogrammed properties that dictate the course of the disease are related to the Warburg effect, the acidification of the TME, the mutations that alter the availability of certain amino acids, hypoxia, the metabolite-sensing-induced mechanisms, the macromolecules and organelles released in the TME and the reverse Warburg effect.

#### 1.1.1. Warburg Effect

Even in the presence of oxygen and fully functioning mitochondria, tumor cells preferentially use aerobic glycolysis instead of oxidative phosphorylation, which supports the production of building blocks, reductive power and ATP for proliferating cells, but it has lower ATP yield per molecule of substrate, a phenomenon termed the Warburg effect [31]. This metabolic adaptation is regulated by a number of oncogenes and tumor suppressor genes, such as PI3K/Akt/mTOR signaling pathway, C-MYC and hypoxia inducible factor (HIF) [32].

Glucose and glutamine are the main substrates of cancer cells; thus, their uptake is increased in cancer. Glutamine metabolism is mainly driven by the C-MYC oncogene, a proto-oncogene often constitutively overexpressed in cancer, which increases the expression of glutamine transporters and their metabolic enzymes [21,27] (Domblides et al. 2019). C-MYC, also promotes activation of glycolytic genes and glucose transporters [33]. In this regard, cancer cells often overactivate mTOR, which upregulates the translocation of the glucose transporter GLUT1 to the plasma membrane [27]. PI3K/Akt/mTOR pathway activity is frequently upregulated in cancer as it is involved in the regulation of cell proliferation, growth, cell size, metabolism, and motility [21].

In cancer cells, hypoxia triggers a metabolic shift towards glycolysis since the absence of oxygen limits the use of OXPHOS [34]. Hypoxia conditions also induce the overexpression of several glycolytic enzymes through Akt activation [35]. Hypoxia inducible factor Alpha (HIF-1α) transcription factor targets genes that constitute an adaptation for metabolic rewiring. These include vascular endothelial growth factor (VEGF) and its receptors to regulate vascular remodeling and plasticity, enzymes of the glycolytic pathway (i.e., hexokinase 2, lactate dehydrogenase) and glucose transporters, as well as Carbonic Anhydrase IX (CA-IX) for pH regulation [36].

#### 1.1.2. Acidification of the TME

As per the Warburg effect, cancer cells produce significant amounts of lactate that accumulates in the TME. Lactate is exported by co-transport with protons through the Mono-Carboxylate Transporters (MCTs) and promotes acidification within the TME. Additionally, acidification can be potentiated by membrane CA-IX upregulated by the HIF pathway. CA IX promotes acidification in the TME since its active site faces the extracellular space and catalyzes CO_2_ hydration, which produces protons outside of the cancer cells. CA IX also cooperates with bicarbonate transporters, as well as MCTs, to transport acid from the intracellular space of the cancer cells to the TME [37]. The proteoglycan-like domain of CA IX mediates non-catalytic export of protons coupled with export of lactate in cancer cells through MCT [38].

Acidification of the TME provides a growth advantage to tumor cells at the expense of immune cells, as it inhibits proliferation and function of T cells, affects the function of monocytes and NK cells, and acts over the chemotaxis and migration of neutrophils and DCs while promote Treg formation, myeloid-derived suppressor cells (MDSCs) and M2 polarized macrophages infiltration with greater immunosuppressive effects on T cells [30] (Figure 3).

#### 1.1.3. Role of Amino Acids in Battle between Immune and Cancer Cells

Changes that disrupt the amino acids metabolism and availability in the TME constitute important targets for cancer cells to circumvent the immune response since immune cell correct functioning requires homeostasis of the amino acid metabolism. For example, Indoleamine 2,3-Dioxygenase (IDO) expression can be induced by Interferon-gamma (IFNγ) or can be constitutively overexpressed by mutations in *BIN* and *KIT* oncogenes [27]. IDO regulates the tumor functions associated with inflammation and its overexpression leads to tryptophan deprivation, which suppresses CD8+ T effector cells and NK cells while inducing Tregs and MDSC, impairing immune cell functions (Figure 3) [27]. Furthermore, overexpression of glutaminase leads not only to depletion of glutamine levels but also to high glutamate levels that might impair the immune function [28]. Cysteine is necessary for glutathione synthesis, ROS detoxification, and T-cell activation [39] (Siska et al. 2016). Thus, inhibition of cysteine uptake impairs T-cell activation, as it is critical for the Reactive Oxygen Species (ROS) detoxification machinery [28].

#### 1.1.4. Hypoxia

Hypoxia is a common feature of tumor tissues that arises because of oxygen diffusion limitations and an abnormal vasculature. Response to hypoxia is mediated by HIF-1α transcription factor which is commonly overexpressed in tumors [40]. HIF-1α can be activated by growth-signaling pathways, such as PI3K/Akt/mTOR or MAPK [41], and some oncometabolites, such as fumarate and succinate, can also induce HIF-1α signaling [42].

HIF-1α promotes the expression of several cytokines and chemokines that attract monocytes, macrophages and myeloid cells [43]. Monocytes differentiate into tumor-associated macrophages (TAMs) whose infiltration is closely related to tumor cell proliferation as they impair T-cell proliferation and cytotoxic activities, trigger inflammation and promote cancer; meanwhile, myeloid cells, such MDSCs, contribute to immunosuppression [44]. O_2_-deprived cancer cells may also release ROS and metabolites, such as adenine and lactate, that inhibit T-cell function and recruit Tregs with immunosuppressive functions [28]. The 5′-nucleotidase pathway is induced by hypoxia leading to adenosine accumulation in tumors impairing the immune function (T cells, NK and activation of immunoregulatory M2) [28].

Together with HIF-1α (HIF1A), at least five additional members of the HIF family: HIF-1β (ARTN), HIF-2α (EPAS1), HIF-2β (ARNT2), HIF-3α (HIF3A), have been discovered in human. HIF-1β is a dimerization partner of HIF-1α for the hypoxia signaling that together with HIF-1α form the transcriptional active complex HIF-1. HIF-1β is constitutively expressed and unaffected by hypoxia. However, certain tumor cell lines derived from different entities are capable to elevate HIF-1β expression under hypoxic conditions which implies a survival benefit. It has been demonstrated that HIF-1β is a potential direct target gene of HIF-1 in human Hep3B hepatocellular carcinoma cells [45]. In the case of HIF-2α, it has been found that is involved in the extracellular ATP promotion of breast cancer invasion and epithelial–mesenchymal transition [46]. Overexpression of HIF-2β is associated with decreased cell proliferation and better prognosis in gastric cancer [47], whereas HIF-3α is a potent prognostic biomarker in different kinds of cancer [48].

#### 1.1.5. Signaling Events Induced by Metabolite-Sensing

Cells need to perceive the changes in intracellular and extracellular metabolites to interact with the environment and respond accordingly. Therefore, in addition to their role in biosynthesis, metabolites also modulate protein activity, cell signaling, and gene expression. Thus, metabolic signals contribute to immune cell function and impact anticancer immunity so they can be modulated to optimize cancer [49]. AMP-activated protein kinase (AMPK) and mTOR signaling are master regulators of cell metabolism and constitute good examples of the signaling pathways induced by metabolic-sensing. AMPK is a sensor of cellular energy and nutritional status; therefore, it plays a key role in the regulation of the cell energy homeostasis and carcinogenesis as energy balance dysregulation is an important driver of the alterations in cancer [50]. Changes in AMP/ATP ratio regulate the activity of key metabolic enzymes governing anabolic and catabolic pathways that facilitate cell survival, control mitochondrial respiration, nutrient transport, autophagy, differentiation, and cell polarity [51]. An increased ratio of AMP/ATP in metabolically stressed cells enhances phosphorylation of AMPK that results in activation of ATP- producing pathways [21]. Moreover, mTORC1-mediates the amino acid sensing with the help of arginine sensor proteins and the arginine level controls mTOR activity [50]. In cancer cells dysregulation of the PI3K/Akt or the Ras/ERK signaling are coupled to mTOR activation which controls differentiation, proliferation, survival, cytoskeleton organization and autophagy [51]. Other examples of metabolite sensors constitute transmembrane proteins, such as G-protein-coupled receptors, that can function as a succinate and α-ketoglutarate receptor that sense metabolites for the TCA cycle [50] which importance in oncogenesis relays in the control of cellular energy and synthesis of precursors for biosynthetic pathways [52]. Metabolic signals contribute to immune cell function and impact anticancer immunity, so that they can be modulated to optimize cancer immunotherapy. New cancer therapies can be found by better understanding the nutrient-sensing processes in T cells, since it will allow us to enhance their metabolic capability to compete for nutrients with cancer cells. Immunometabolic signaling is dynamically regulated through the interplay of nutrient signaling networks and serine/threonine kinases, such as the PI3K–AGC kinases, mTOR and LKB1–AMPK pathways [48]. Changes such as activation of AMPK or inhibition of mTOR promote Tregs over TEFFs and inhibit the function of NK cells [53]. Phospholipids can also be key second messengers that are highly regulated regarding to turnover, since they influence downstream immunometabolic pathways; for example, PI3K activity induces diverse signaling pathways involved in regulating cellular function, including Akt, phosphoinositide-dependent protein kinase 1 and mTOR. Lipid molecules such as cholesterol and FAs play a key role in the activation, differentiation, and function of T cells [54] (Wei et al. 2017). PI3K also controls lipid and cholesterol content which are integral components of cellular membranes and is also an important regulator of autophagy essential for T-cell homeostasis, function and differentiation [49] (Saravia et al. 2020).

Glucose sensing regulates and shapes T-cell responses as glucose transporting GLUT1 expression and glycolysis promote TEFF cell and Treg cell proliferation impair Treg cell lineage stability and suppressive function [54] (Wei et al. 2017). T and tumor cells reprogram their metabolism to enhance glucose uptake and aerobic glycolysis to compete for glucose in the TME and in the glucose-limited TME, the immune response of tumor-infiltrating T cells is less effective than in T cells from glucose-rich environments [54]. Activated T cells have a glucose-sensitive metabolic checkpoint controlled by AMPK, which regulates mRNA translation and glutamine-dependent mitochondrial metabolism needed to sustain T-cell metabolic homeostasis mediating adaptive immunity [55].

Numerous studies have demonstrated that drugs that inhibit the mechanistic target of rapamycin (mTOR) and activate AMP-kinase (AMPK) have beneficial effects in promoting differentiation and blocking proliferation of different cancers, i.e., in hematological malignancies such as acute myeloid leukemia (AML) [56]. In solid cancers such as glioma, agents such as isogambogenic acid [57] and compound C [58] inhibit glioma growth through the activation of the AMPK–mTOR pathway. In pancreatic cancer, repurposing of metformin and aspirin by targeting AMPK–mTOR and inflammation is used for prevention and treatment [59]. In prostate cancer it has been found that small-molecule natural inhibitors of the PI3K/Akt/mTOR Pathway can be useful for chemoprevention and intervention [60]. In breast cancer, AMPK activators suppress breast-cancer-cell growth by inhibiting DVL3-facilitated Wnt/y can be useful for chemn by targetin [61]. In non-small-cell lung cancer (NSCLC) heat treatment led to the increased phosphorylation of AMPK and the decreased phosphorylation of mTOR in vitro and in vivo. Moreover, in NSCLC, the GRP78 knockdown inhibits the AMPK–mTOR pathway, and the AMPK inhibitor compound C decreased heat-induced autophagy, suggesting that activation of ER stress was involved in autophagy induction and promotion of the AMPK–mTOR pathway [62].

#### 1.1.6. Macromolecules and Organelles Released in the TME

Macromolecules and organelles released by noncancerous cells in the TME support biosynthetic and bioenergetic needs of the cancer cells and due to the overlapping metabolic demands of cancer and immune cells in the tumor ecosystem, the transfer of this metabolic resource confer cancer cells with a growth advantage over immune cells [63]. Intercellular organelle transfer has been demonstrated to be an important survival mechanism under external stress conditions [63]. Being either unidirectional or bidirectional, intercellular organelle transfer is a process in which whole organelles are donated from one cell to another with the transfer of cytosol and plasma membrane components, as well as of small molecules and ions by ATP-dependent mechanisms through nanotubes. In this process, the endoplasmic reticulum/Golgi bodies, endosomes, lysosomes and mitochondria originating in one cell (organelle donor) are transported to another cell [63]. Mitochondrial transfer phenomenon is involved in cancer progression by modulating inflammation processes, chemotherapy resistance and, thus, cancer cell survival, and it can be advantageous for the survival of cancer cells that have fully functioning mitochondria [64].

#### 1.1.7. Reverse Warburg Effect

The novel concepts of the “reverse Warburg effect” and the “autophagic tumor stroma model of cancer metabolism” are supported by the evidence that enhanced aerobic glycolysis and/or autophagy in the cancer-associated fibroblasts (CAFs) sustains epithelial cancer cell growth via the secretion and transfer of high-energy metabolites by the tumor stroma including ketones, lactate, amino acids (glutamine) and nucleotides [65]. Additionally, CAFs contribute to a strong immunosuppressive effect having lactic acidosis in the TME [66] and CAF-derived exosomes have also been shown to contain nutrients [65,67].

### 1.2. Crosstalk between the TME, Extracellular Matrix and Cell Metabolism in Cancer

Cancer cells interact with both the immune system and the stroma, while fibroblasts, and other stromal cells, can interact with immune system and influence its response. A deep comprehension of the dynamic interactions between cancer stromal and immune cells in the TME ecosystem is required to predict the resulting pro- or antitumor effects [2]. The extracellular matrix (ECM) is a complex network of secreted proteins that creates a complex 3D microenvironment providing mechanic support for tissues and organs while controlling many cell functions, including cell polarity, migration, proliferation, oncogenic transformation, metabolic plasticity and responsiveness to therapies targeting cell metabolism [68]. CAFs and cell–ECM interactions are key elements in controlling these metabolic changes. CAFs and other stromal cells can recruit immune cells to the tumor [2] and CAFs undergo the reverse Warburg effect providing cancer cells with glycolytic metabolites that help cancer cells support the Warburg effect [69]. ECM components, such as fibronectin and laminin, are internalized by integrins and control nutrient signaling pathways in a reversible control manner which affects the ability of cancer cells to grow under nutrient deprived conditions and this way have a metabolic advantage over the immune cells [70].

## 2. Genome Scale Metabolic Modeling in Cancer

### 2.1. GEMs

GEMs are mathematical representations of the metabolic potential of an organism; they define the whole set of stoichiometry-based, mass-balanced metabolic reactions in an organism. To date, a number of generic GEMs of human metabolism have been reconstructed, including Human Metabolic Reaction (HMR) series: HMR [71] and HMR2 [72]; The Recon series: Recon 1 [73]; Recon 2 [74] and Recon3D [75]; and the Edinburgh model [76] and they have been extensively used in the study of cancer metabolism (Table 1). A key component of GEMs is the biomass function, an artificial reaction that serves as a surrogate for the metabolic demand (i.e., building blocks, metabolic energy and reductive power) required for growth and proliferation. A second feature of GEMs are Gene-Protein-Reaction (GPR) annotations, a set of rules that define the isoenzymes or protein complexes that catalyze each reaction, enabling to map transcriptomics and proteomics measures to GEM reactions [77,78,79,80].

### 2.2. Constraint-Based Modeling

**Figure 4:**
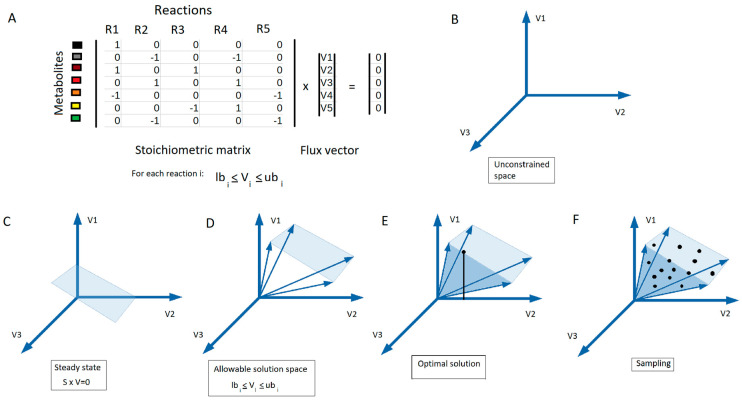
Genome-scale metabolic modeling. (**A**) FBA optimization maximizes a linear objective function, ψ(*v*), formed by the dot product of the flux vector containing each reaction flux, (*v_i_*) by a predetermined coefficient, *c_i_*, in the stoichiometric matrix (*S*) subject to a steady-state assumption, *Sv* = 0, as well as lower and upper bounds on each reaction flux (*lb_i_* and *ub_i_*). From the unconstrained space (**B**), the allowable solution space is defined by specifying the steady-state assumption (**C**), as well as lower and upper bounds on each reaction flux (**D**). Various methods can then be used to interrogate the solution space, such as (**E**) optimization for a biologically motivated objective function to identify a single optimal solution (e.g., flux vector in case FBA optimization method is used) or (**F**) sampling to provide an unbiased characterization via flux vectors uniformly distributed in the solution space.

The analysis of the distinct usage of metabolic pathways in cancer metabolism requires the quantification of metabolic fluxes. A metabolic flux is the rate at which substrates are converted to products through a given reaction or pathway. Constraint-based modeling, sometimes referred to as stoichiometric modeling, is a modeling approach that uses a set of linear constraints to simulate steady-state metabolic fluxes across a metabolic network. This approach assumes a metabolic quasi-steady state where no intracellular metabolites accumulate or deplete, as their average concentration can be assumed constant in time. This assumption is generally valid for intracellular metabolites as they transition towards a quasi-steady state several orders of magnitude faster than variations in extracellular metabolite concentrations or the gene-expression program of the cell [89]. Since intracellular concentrations are assumed constant under quasi-steady state, the input and output fluxes for each metabolite must be balanced. In constraint based-modeling, this constraint is written as *S* × *v = 0*, where *S* is the stoichiometric matrix, which defines the stoichiometric coefficients of each metabolite in each of the reactions in the network, and *v* is the vector of steady state fluxes (Figure 4A). Additionally, flux boundaries can be set to satisfy thermodynamic laws (i.e., reaction reversibility) or with measured rates of metabolite uptake and secretion (Figure 4D). Since constraint-based modeling relies primarily on network stoichiometry, which is well-defined on a GEM and does not require precise knowledge of the kinetic properties of enzymes, it is widely used to model metabolic fluxes in GEMs [77,78,79,90].

However, the space of solutions emerging from the abovementioned constraints in a GEM is generally largely undetermined (Figure 4B) (i.e., there is a wide range of possible flux distributions, many of them biologically irrelevant). To overcome this limitation, Flux Balance Analysis (FBA) identifies the optimal flux distribution(s) by maximizing a biologically motivated objective function, such as the flux, through the biomass reaction (Figure 4E) [91]. Nevertheless, there can still potentially be multiple flux distributions that maximize a given objective. Phenotypically different alternate optimal solutions can yield the same optimal objective value resulting from a FBA optimization. The range of optimal flux distributions can be estimated by Flux Variability Analysis (FVA). FVA estimates the allowable range of flux values through a given reaction by finding the maximum and minimum possible fluxes from the several solutions that satisfy the optimal objective function’s value, such as maximum biomass production, called the alternate optimal solution space [92]. Therefore, alternate optimal solution space can be quantified by using FVA, since it provides a range of minimum and maximum values for each reaction of the system [92].

An alternative to these methods consists of using sampling methods that avoid the need to specify an objective function by estimating the distribution of possible flux values in the solution space through techniques such as Uniform Random Sampling and Hit-and-Run Sampling [93,94,95,96]. The solution space can then be characterized statistically from the set of sampled flux vectors in terms of a probability density function (Figure 4F). Uniform Random Sampling obtains a statistically meaningful number of solutions uniformly distributed through the entire solution space. Hit-and-Run Sampling is a Markov chain Monte Carlo method in which the solution space is randomly sampled [97].

Given that the solution space is often underdetermined, recent studies have enabled the incorporation of thermodynamics constraints and integrated metabolomics data to ensure that the simulated flux maps are thermodynamically and kinetically feasible. For example, anNET, by testing the thermodynamic consistency of the data by identifying thermodynamically infeasible errors and by predicting the concentrations beyond the quantitative data being measured, makes a quality check of metabolite concentrations and enables to identify the reactions whose metabolic flux is actively regulated [98]. Thermodynamics-based Flux Analysis (TFA) integrates quantitative metabolomics data and standard Gibbs free energy release of reactions [99] to add a thermodynamic constraint to each reaction that ensures that the directionality of each reaction is feasible given the ratio of concentrations between substrates and products [100]. While pyTFA and matTFA were the first implementations of the TFA [101], a modified version of matTFA that considers alternative parameter values and methods has been developed in by Tomi-Andrino et al. [102], and recently, mod-matTFA toolbox was implemented.

TC-iReMet2 is a constraint-based modeling approach to account for magnitude of flux changes that combines relative metabolite and transcript time-course data to estimate fluxes, providing a more accurate explanation of flux rerouting over time [103]. Linear Bound Flux Balance Analysis (LBFBA) uses transcriptomic or proteomic expression data and adds on individual fluxes soft constraints whose parameters are estimated from a training expression and flux dataset that are then used to predict metabolic fluxes from expression data in other conditions [104]. Relative Expression and Metabolomic Integration (REMI) uses GEMs to translate differential gene expression and metabolite abundance data into differential fluxes to analyze the dysregulated physiology for any given pair of conditions by integrating differential gene expression and differential metabolite abundances with thermodynamic data into a single framework, and then maximizing the consistency between them [105].

### 2.3. Context-Specific GEMs

**Figure 5:**
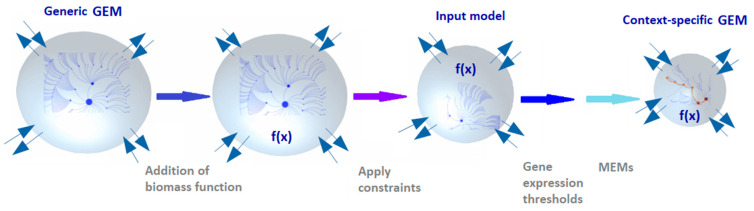
Overview of key steps in the constraint-based modeling process. Extracting a context-specific model representing the metabolism of a tissue type or cell line requires several steps. The most salient steps consist of choosing the kind of generic model to use, the addition of the biomass function, how to constrain the uptake and secretion fluxes in the model, defining of the gene expression threshold to include a gene in a reaction and choosing the MEM algorithm to use for extraction.

At a given cell or tissue, only a subset of enzymes is expressed; thus, it is generally recommended to integrate transcriptomics, proteomics or other condition-specific data to generic GEMs of human metabolism to build context-specific GEMs. A context-specific GEM is a subset of the generic GEM in which inactive reactions are removed to reflect cell-type- or tissue-specific metabolic phenotypes. This contextualization process (Figure 5) depends on: generic GEM chosen, definition of the biomass function, uptake and secretion flux constraints, gene expression levels, metabolomics, and tissue- or cell-specific metabolic functions of the cell or tissue type that need to be active in the extracted model. Additionally, the algorithm and parameter selection (e.g., gene expression thresholds and metabolic constraints) can affect model content and predictive accuracy. Thus, context-specific modeling often requires evaluation procedures for the selection of such parameters [106]. Model extraction methods (MEMs) are omics data-integration algorithms used to create context-specific GEMs. MEMs include GIM^3^E [107], iMAT [108], MBA [109], INIT [82], mCADRE [110], tINIT [82], CORDA [111], FastCore [112] and FASTCORMICS [113]. In all of these methods there are two critical points: (1) the gene expression pre-processing controlling the distinction between active and inactive genes and (2) Gene to reaction mapping by GPR associated with each reaction.

Context-specific GEMs have a wide range of applications, such as identifying putative drug targets [114], predicting of host–pathogen metabolic interactions [115,116] or characterizing the reprogrammed metabolism of liver cancer stem cells [117]. Some databases with published context-specific GEMs include the BioModels Database [118] (https://www.ebi.ac.uk/biomodels-main/ [Accessed on 1 January 2020]), metabolic atlas [119] (https://www.metabolicatlas.org/ [Accessed on 1 January 2020]) and BIGG models database [120] (http://bigg.ucsd.edu/ [Accessed on 1 January 2020]).

### 2.4. Kinetic Models

Constraint-based approaches make use of network stoichiometry to characterize the intracellular fluxes at steady state and cannot generally simulate fluxes and metabolite concentrations outside of the quasi-steady state nor simulate the regulatory loops emerging from the kinetic properties of enzymes. Kinetic models use mathematical expressions referred to as kinetic laws or rate equations which compute the rate through an enzyme-catalyzed reaction as a function of metabolite and enzyme concentrations and the kinetic parameters of the enzymes. Substituting the rate equations in the mass balance equation (*S* × *v*) results in a system of ordinary differential equations (ODEs) describing the metabolite concentration changes over time. The equations for the reaction rates are functions of enzyme concentrations, kinetic parameters and metabolite concentrations, so it is possible to describe the stoichiometric relation between substrate and products considering the enzymatic mechanisms and different levels of regulation (i.e., allosteric or post-transcriptional regulation), making these methods appropriate for multiple omics data types’ integration. Therefore, solving such models enables dynamic analysis of biological systems and allows quantitative predictions of the cells’ states along time for enhanced in silico hypothesis generation. Kinetic models have been typically used to generate efficient strain optimization algorithms [121], although they have also been extensively applied to model cancer metabolism [122,123,124].

Kinetic models are typically built in a bottom-up manner: for each reaction, a kinetic law with its respective parameter values should be provided, resulting in model structures with great amounts of parameters. The model parameters can be experimentally assessed through the literature, experimental measures or databases, such as Brenda [125] or SABIO-RK [126]. Alternatively, model parameters or parameter uncertainty can be estimated by using parametric estimation methods [127,128] or Monte Carlo methods [129,130].

Kinetic models have to be compatible with physicochemical laws, such as electro-neutrality and osmotic balance, experimental flux and metabolite measurements to reduce model uncertainty and discard the reaction directionalities that are not consistent with the observed physiology [131]. Kinetic rate laws should also be able to model enzyme saturation and the mechanistic regulatory details [131].

Because of this complexity, kinetic models are often of limited scope, covering one or a few metabolic pathways, but the network of metabolism is highly interconnected and the dynamics of the whole system are required to simulate its behavior. Recent efforts have been made towards building genome-scale kinetic models [132,133,134,135].

Alternatively, it is possible to reduce the model complexity by modeling in detail only the kinetics of reactions that are important for a particular physiological condition and for the remaining reactions use simple approximate kinetic laws, such as considering a quasi-steady state, and then modeling these parts by stoichiometry only. Ensemble modeling wherein multiple models are combined in order to create a feasible solution space constitutes a solution to address kinetic parameter scarcity and small-scale preference [136,137]. ORACLE [138], GRASP [139] and K-FIT [140] are packages for ensemble modeling.

### 2.5. Application of Metabolic Analysis Tools in Cancer Research

#### 2.5.1. Software for Constraint-Based Modeling Tools

One of the most widely used frameworks for constraint-based modeling is the family of Constraint-Based Reconstruction and Analysis (COBRA) packages. Available in MATLAB [141], Python [142] and Julia [143], this family of open-source packages provide community-contributed modules to perform a variety of constraint-based modeling analysis, including FBA, FVA, flux sampling, in silico gene Knockouts (KOs) and a variety of MEMs algorithms that allow network integration of metabolomics, transcriptomics, proteomics and thermodynamic data. The MEM data integration is used to simulate different metabolic phenotypes, including growth rate and gene essentiality. Essential and synthetic lethal genes are defined by using in silico KO strategies that disrupt the biomass reaction’s flux by blocking the biosynthesis of at least one essential metabolite and halt cellular proliferation. Other frameworks for constraint-based modeling and/or GEM metabolic reconstruction are reviewed in detail in [141,144] (Table 2).

#### 2.5.2. Application of GEMs in Metabolic Cancer Research

GEMs have been widely used to analyze the metabolic phenotype underlying cancer, find cancer-specific metabolic essential genes that are putative novel drug targets in cancer and stratify patients [169]. Table 3 describes a number applications of GEMs in cancer research, such as modeling of the Warburg effect; predicting potential biomarkers, drug targets and adverse drug effects; identifying metabolic signatures for drug repositioning; and determining and identifying anti-metabolites for metabolic inhibitors to co-administrate in adaptive therapies.

#### 2.5.3. Modeling the Metabolic Crosstalk between Cell Populations

To model the metabolic crosstalk between multiple cell populations, such as substrate competition or cross-feeding (i.e., the metabolic products of a cell population are used as substrates for a second cell population), multiple GEMs, each representing a subpopulation, can be coupled (i.e., connected to a shared extracellular compartment). This approach has been pioneered in the modeling of bacterial communities, where metagenomic sequencing data are used to build models of specific bacteria species present in the community, which are subsequently coupled [183]. This has enabled modeling the metabolic host–microbiome interactions in the human gut and their role in health and disease [184,185]. Additionally, 32 organ and cell-type-specific GEMs have been integrated to build physiologically constrained female and male whole-body metabolic models that in addition of predicting how the microbiome may modulate human metabolism, they can also predict organ metabolic essentiality, biomarkers of inherited metabolic diseases and inter-organ metabolic cycles [186]. However, modeling the crosstalk between cancer and immune population in the cancer stroma is a major challenge due to the intermingling of the different cell populations, to the tumor heterogeneity and to the dynamics changes happening both in cancer and in the immune populations. Building a context-specific GEM by using bulk transcriptomics, proteomics or metabolomics will result in a single model representing the average profile of the tumor, and, thus, it will be unable to capture metabolic heterogeneity, nor the metabolic crosstalk between cell populations. However, thanks to the use of single-cell RNA-Seq (scRNA-Seq) is possible to analyze the composition and the population changes. For example, thanks to the use of scRNA-Seq, Wu et al. [187] discovered rare cell types in non-small-cell lung cancer (NSCLC) such as follicular dendritic cells and T-helper 17 cells. Lin et al. [188], using scRNA-Seq, discovered in pancreatic ductal adenocarcinoma (PDAC) distinct cell types in primary and metastatic PDAC tissues including tumor cells, endothelial cells, cancer-associated fibroblasts (CAFs) and immune cells. Cancer cells have higher inter-patient heterogeneity, whereas stromal cells are more homogenous across patients. Furthermore, they found that the expression levels of cell-type-specific markers for EMT+ cancer cells, activated CAFs and endothelial cells significantly associated with patient survival. Moreover, in PDAC, Dominguez et al. [189] identified a population of CAFs that are programmed by TGFβ and express the leucine-rich repeat containing 15 (LRRC15) protein. These LRRC15+ CAFs surround tumor islets and are absent from normal pancreatic tissue. The integration of the information provided by scRNA-Seq with GEMs has led to the development of approaches such as popFBA *or* single-cell FBA.

##### PopFBA

PopFBA [190] is an extension to FBA to study the cooperating metabolism through the exchange of a defined set of metabolites within the presence of several subpopulations in a population sharing a common environment. Spatial proximity dictates differences of the distinct components in the tumor for nutrient exchange with the plasma and other cells within the populations. A single GEM is used as a building block, and the population model consists of multiple clones with identical stoichiometry, constraints and sharing the plasma nutrient supply. Linear programming optimization is used to find the optimal growth rate of the entire tumor mass, allowing us to investigate the cooperation among different clones and the different metabolic strategies taken. However, because all the models share the same stoichiometry, this approach is unlikely to encapsulate the metabolic differences between distinct cancer and immune cell populations. Therefore, popFBA only allows us to explore how metabolic heterogeneity and cooperation phenomena affect the overall growth of cancer cell populations.

##### Single-Cell FBA (scFBA)

It is possible to add constraints on the single-cell fluxes by using single-cell transcriptomes derived from scRNA-Seq experiments. Coupling the information of scRNA-Seq with extracellular fluxes within the FBA steady-state modeling framework allows us to obtain feasible solutions for the prediction of intracellular fluxes. ScFBA [191] takes as input a template metabolic network map and an scRNA-Seq matrix. It also allows us to employ the information on bulk expression profiles. Single-cell fluxomes and possible metabolic interactions among them are predicted by using scFBA, starting from bulk extracellular fluxes and a multiscale model with scRNA-Seq that optimize an individual biomass function to identify the possible combination of single-cell steady states given the constraints on scRNA-Seq and extracellular bulk fluxes. The flux distribution of each reaction and each cell interacts with other cells in the population via release/uptake of metabolites into/from the TME. ScFBA first creates a multiscale population model: for each single cell in the bulk, the same stoichiometry of the metabolic network map is first used, and then the constraints on the fluxes of the individual networks are integrated by assigning flux boundaries as a function of the expression state proportionally to the activity score of that reaction in each cell. This procedure is implemented without creating context-specific models from generic ones. Finally, each single cell in the bulk is represented by a single-cell compartment of the multiscale model. Thus, each single-cell compartment has different metabolic parameters and allows cooperation between cells, as well as the flow of metabolites between the TME and the cells.

## 3. Discussion

The immune system is critical in fighting cancer, as the immune response can lead to the destruction of cancer cells. Immune cells and cancer cells compete for nutrients within the TME. Some cancer cells gain mutations and change their characteristics to evade the immunosurveillance by several mechanisms. Cancer cells can reprogram their metabolism to enhance proliferation, migration and invasion of distant tissues. Many of these metabolic changes result in an increased uptake of nutrients from the TME, limiting the availability of nutrients for the cells from the immune infiltrate. Knowledge about the utilization of reprogrammed metabolic mechanisms requires the quantification of metabolic fluxes, and GEMs allow us to make computational predictions of the metabolic fluxes of cancer cells. Modeling metabolism at the genome-scale requires us to reverse-engineer the network structure, and then to add stoichiometric, thermodynamic and/or enzymatic capacity constraints on the network. Using transcriptomic and proteomic data, we deem that it is possible to identify the presence of the enzymes that catalyze the active reactions in a given cell and tissue type. Metabolomic data can be used to determine the rate at which metabolites are produced or consumed from the extracellular medium (i.e., extracellular fluxes) and can have a significant effect on predictions [191]. Extracellular fluxes can be measured ex vivo with experiments that have a controlled extracellular medium. Indeed, culturing cells from human biopsies from which transcriptomic or proteomic data are obtained and measuring metabolite concentrations in spent medium constitute a first attempt towards cancer personalized medicine [192]. To test therapies that remain in the preclinical stage, while minimizing the possible toxicity risks, if patient consent is available, it is possible to use a tumor sample for in vitro manipulation with primary cell culture or organoids or even subcutaneously transferring into mice or zebrafish for growth to obtain patient-derived xenografts in which we can test different drugs and concentrations [193].

In this work, we reviewed the state-of-the-art in modeling the tumor metabolism at the genome-scale and the crosstalk between cancer and immune cell metabolism in the context of the existing framework for constraint-based modeling and/or genome-scale metabolic reconstruction, including a number of approaches involving the application of GEMs. We also addressed the population’s heterogeneity problem, which is that the actual cellular population composition is undetermined, and thus, the average values do not describe the population fluxes well; therefore, an additional layer of complexity must be modeled by using specific methods. We posit that such a framework has yet untapped potential to model the metabolic crosstalk of cancer cells and immune cell populations that plays a key role in cancer progression and could potentially pave the way for a new generation of therapies that potentiate antitumoral immune response.

The heterogeneity of the tumor metabolic reprogramming is not fully understood, as there are experimental limitations for the metabolomics studies, in that, although they are able to provide insights into the cellular metabolic status of cells, there is still a lack of information regarding the cause or effect of metabolite changes and in silico models. Integrating multi-omics data may provide a broader picture, thus enabling the identification of the regulatory mechanisms underlying in the pathophysiology of complex diseases, such as cancer [194]. The TME constitutes a complex adaptive ecological system, and due to the difficulty in modeling the interactions between multiple cell types modeling, the TME remains challenging. Capuani et al. [195] reconstructed a large-scale in silico metabolic model of interacting human CAFs and tumor cells for the lactate shuttle. Shan et al. [196] implemented a multiscale modeling approach to interrogate the implication of the TME on the Warburg effect, the reverse Warburg effect and glutamine addiction. With the use of the ScFBA algorithm developed by Damiani et al. [191], it was possible to model a heterogeneous cancer cell population and represent the metabolite exchange between different cell types. Because TAMs can be induced by oncometabolites that activate in macrophages’ cancer-promoting pathways, reversing the polarization of protumoral TAMs is likely to be an effective strategy against cancer [197]. In this direction, a new avenue constitutes to interconvert pro-inflammatory M1 (with the ability to metabolize arginine to nitric oxide) to wound-healing M2 (with the ability to metabolize arginine to ornithine) [198] populations of macrophages by using approaches such as depletion of TAMs, reprogramming of TAMs toward M1 polarized macrophages, acting over the recruitment of monocytes into the tumor or inhibiting the endothelial–mesenchymal transition [199]. In this line, Li et al. [199] used an interaction network of cancer cells and macrophages to investigate how the polarization of macrophages can interact with the epithelial–mesenchymal plasticity of cancer cells. This study suggested that therapies against malignancy that enhance M1 domination and cancer-free steady-state require mechanisms that synergistically promote the transition of mesenchymal to epithelial cells and keep a low growth rate of mesenchymal cells.

The TME and the ECM affect tumor cell metabolism, control cell polarity, migration, proliferation and oncogenic transformation; consequently, it is essential to understand the contribution of nutrient transit from the TME to support cancer cells’ growth under nutrient-starvation conditions [70]. CAFs represent a significant proportion of the TME, and they have been shown to contribute to tumor growth, metastasis and resistance to therapy through the regulation of cancer metabolism, as they secrete metabolites and generate a more fibrotic ECM [70]. Integrins are plasma membrane receptors that mediate cell–ECM interactions. Modeling the crosstalk between CAFs and cancer cells, integrin trafficking, nutrient signaling and different mechanisms through which cancer cells can exploit different nutrient sources from the TME will enormously contribute to understanding the metabolic plasticity and responsiveness and resistance to therapy of the cancer cells.

Future developments will likely involve methodological and translational advancements, such as using richer datasets with additional data sources from different cell types from scRNA-Seq and clinical samples that include the information of cellular regulatory mechanisms or modeling interactions of cancer cells with the surrounding cells from the TME, as well as predicting and circumventing the drug resistance of metabolic cancer drugs by building integrated kinetic and stoichiometric models of cancer metabolism. For example, The Cancer Genome Atlas (TCGA) database (https://www.cancer.gov/tcga [Accessed on 1 January 2020]) is a good resource of cancer high-throughput-omics data that can be collected and integrated to holistically perform analysis, using GEMs. Outside of cancer, these bottom-up systems’ biology approaches that drive drug repurposing and drug discovery can be used to identify therapies for other diseases [200].

## 4. Conclusions

Expanding the understanding of the dynamic interplay of numerous cell types, such as cancer cells, immune cells and stromal cells within TME is likely key to develop effective cancer therapies. Cancer cells reprogram their metabolism to capture substrates needed to sustain proliferation with increased avidity and produce toxic metabolic byproducts, inhibiting immune cells’ function and promoting immune evasion. Thus therapies selectively targeting the metabolic crosstalk between cancer and immune cells could potentially restore immune function within the tumor, leading to improved therapeutic outcomes. In this regard, the GEMs approaches reviewed in this work are promising tools to analyze the metabolic crosstalk underlying immunosuppression in the TME, stratifying patients, identifying key players promoting immunosuppression in each patient strata, and repositioning drugs to target them.

## Figures and Tables

**Table 1:** Human metabolic generic models and their complexity in terms of number of genes, reactions and metabolites. Adapted from [79].

Human Generic Model	No. of Genes	No. of Reactions	No. of Metabolites	Cancer-Type Application	Application References
Human Metabolic Reaction (HMR) [71]	3668	8100	6000	Renal carcinoma	[81]
Human Metabolic Reaction (HMR2) [72]	3765	8181	6007	Hepatocellular carcinoma	[82]
Recon 1 [73]	1496	3311	2766	Generic cancer	[83,84,85]
				16 cancer types	[82]
				NCI-60 CCLs	[86]
Recon 2 [74]	1789	7440	2626	9 cancer types from TCGA	[87]
Recon 3D [75]	3288	13,543	4140	Prostate cancer	[80]
Edinburgh model [76]	2322	2823	2671	Colon and breast CCLs	[88]

Abbreviations: (CCL) cancer cell line.

**Table 2:** Software applications for constraint-based modeling and/or genome-scale metabolic reconstruction. In References [141,144], all of these tools are reviewed in more detail.

Name (and Reference)	Language	Interface	Development	OS
COBRA Toolbox [141]	MATLAB COBRA.py, COBRA.jl (Python and Julia)	Script	Open source	All^M^
RAVEN [145]	MATLAB	Script	Open source	All^M^
CellNetAnalyzer [146]	MATLAB	Script/GUI	Closed source	All^M^
FBA-SimVis [147]	MATLAB/Java	GUI	Closed source	Windows
OptFlux [148]	Java	Script	Open source	All
Sybil [149]	R	Script	Open source	All
CBMPy [150]	Python	Script	Open source	All
SurreyFBA [151]	C++	Script/GUI	Open source	All
FASIMU [152]	C	Script	Open source	Linux
FAME [153]	Web-based	GUI	Open source	All
PathwayTools [154]	Web-based	GUI/script	Closed source	All
Kbase [155]	Web-based	Script	Open source	All
AutoKEEGRec [156]	MATLAB	Script	Open source	All^M^
AuReMe [157]	Python	GUI	Open source	All
CarveMe [158]	Python	Script		
MetaDraft [159]	Python	Script	Open source	All
ModelSEED [160]	Web-based	GUI	Open source	All
PathwayTools [161]	Common Lisp	GUI (API)	Open source	All
Merlin [162]	Java	GUI	Open source	All
CoReCO [163]	Python	Script	Open source	All
MEMOSys [164]	Java	GUI	Proprietary source	All
GEMSiRV [165]	Java	GUI	Open source	All
MetExplore [166]	Web-based	GUI	Open source	All
RbioNet [167]	Part of the COBRA ToolBox	Script	Open source	All
MetaFlux [168]	Web-based	GUI	Open source (distributed as part of Pathways tools)	All

Abbreviations: (All^M^) all operative systems in which it can run MATLAB, (API) Application Programming Interface, (GUI) Graphic User Interface, (OS) operative system.

**Table 3:** Application of GEMs in metabolic cancer research.

Reference	Category	Concept	Tools Used	Databases	Type of Validation
[170]	CSGEMs to generate metabolic signatures for drug repositioning	PC GEM to explore PC metabolism and repurpose new drugs. Reconstruction performed combining personalized GEMs from individual patient’s transcriptome and PC-specific proteomics data from the HCA.	RAVEN, FastGeneSL [171], DIRAC, TCGAbiolinks, DESeq, gcrma	HPA, HMA, ConnectivityMap2	In silico cell viability assay and in vitro cell assay.
[172]	CSGEMs to predict biomarkers and drug targets	GEM of transcriptional regulator-metabolite associations with mixed computational and wet lab experiments integrating intracellular metabolic profiles of NCI-60 (4) 54 CCLs with transcriptomic and proteomic data. Perform metabolic profiling of CCLs and resolve signaling across multiple regulatory layers.	fitlm (Matlab) sparseNCA	Gene Expression Omnibus NCI-60, HMD, TRRUST, KEGG	In vivo metabolite fold-changes between normal and cancer tissues.
[83]	CSGEMs to predict biomarkers and drug targets	CSGEM to study the role of metabolic alterations for novel therapy targets. Predicts 52 cytostatic drug targets (40% by known drugs). Analyze synthetic lethal drug targets to identify drug synergies.		NCI-60	shRNA data, cytostatic scores for single and double drug target predictions, synergistic drug targets via yeast orthologs.
[173]	GEMs to identify antimetabolites for drug design	Assess anticancer effects of drugs structurally similar to DrugBank [174]. Uses Tanimoto scores from OpenBabel [175] to assess structural similarity between DrugBank drugs and metabolites CSGEM predicted to be essential for maximal growth rate. Developed pyTARG to constrain the HMR, using 34 CCLs and 26 healthy tissue RNA-Seqs. Implemented FBA within PyTARG to quantify the original drug affecting reactions rates decrease. Model the impact of a relative inhibition on global cell metabolism.	OpenBabel [175], pyTARG, COBRApy	DrugBank [174], KEGG, BioProject, HPA, GEO, BioModels	Differential effects of a lipoamide analog on MCF7 and ASM cells. Proof of concept of identification of therapeutic windows.
[109]	GEMs to identify antimetabolites for drug design	Proteomics samples from 27 HCC patients and 83 healthy individuals from HPA to reconstruct cancer and healthy GEMs with the tINIT from the HMR 2.0 generic GEM. CSGEMs to identify antimetabolites used as anticancer drugs. Healthy GEMs to explore candidate antimetabolites toxicity on healthy samples.	RAVEN (gap-filling, tINIT, checkTasks)	HPA	Usage of antimetabolites for treatment of HCC demonstrated by the inhibitory effect of the l-carnitine analog, one of the predicted antimetabolites, on the proliferation of the HepG2 CCL.
[176]	GEMs to identify metabolic inhibitors to administrate with drug combinations in adaptive therapies	Found that taxane-treated breast cancer cells undergo a metastable transition in which they depend more on oxidative and non-oxidative glucose metabolism conferring them resistance to doxorubicin. Predict that these rewired cancer cells can be effectively targeted when a glucose metabolism inhibitor is co-administered with doxorubicin.	Prism (GraphPad)		In vivo experiments with mouse models, patient explant system.
[177]	GEMs to explore cancer metabolism biology	Central C and N RMGEM to study the interplay between glucose and glutamine for biomass formation in ammonia microenvironment. Perform Warburg effect quantitative. Used the RMGEM to do FBA to study all possible glutamine fates. Found that glutamine can supply C sources for cell energy production and can be used as a C and N source to synthesize essential metabolites.	FAME		
[178]	GEMs to explore cancer metabolism biology	HPaA to explore the prognosis of each protein in 17 major cancers. Uses CSGEMs to identify tumor growth involved genes. Based on transcriptomics of ~ 8000 patients with clinical metadata. Revealed that survival is associated with upregulation mitosis and cell growth genes while downregulated genes are mostly involved in cellular differentiation.	Kaplan–Meier plots, PCA	HPaA [178], BioModels, TCGA, GO, GDC	Immunohistochemistry.
[179]	CSGEMs to explore cancer metabolism biology	Merged 374 CSGEMs from the HPaA to reconstruct a generic CRC GEM. Identified the mayor differences between tumor and normal samples in terms of highly perturbed metabolites by applying modules reporter metabolite and reporter subnetworks algorithms. Mayor differences were related to the glutathione, arginine and proline metabolic reprogramming.	PIANO (R) (KEGG and GO enrichment analysis), CRC (Bioprofiler analysis), RAVEN, Kaplan-Meier survival analysis, log-rank *p*-value	HpaA, BioModels, TCGA, GO, GDC	ODC1, SMOX, SRM and SAT validated in vivo and in vitro, using 15 patients and 4 CRC CCLs.
[71]	GEMs to explore cancer metabolism biology	HMR 2.0 and proteomics data in HPA to construct consensus hepatocytes GEM (iHepatocytes2322) that improves previous GEMs including an extensive description of lipid metabolism. GEM used to analyze transcriptomics data from non-alcoholic fatty liver disease patients.	INIT, Reporter Subnetwork analysis checkTasks (RAVEN), RAVEN	HPA, Uniprot, GEO	Hepatocytes biological functions of hepatocyte-specific GEM demonstrated by simulating 256 metabolic functions of HepatoNet, using checkTasks/fitTasks (RAVEN).
[90]	GEMs to explore cancer metabolism biology	GPR called S-GPR considering transcripts stoichiometry. Investigate PC cells metabolic effects chronic exposure to an endocrine disruptor.	Mann-Whitney test, FASIMU, Gimme, Mat, pFBA MADE	HMD, Lipid Maps, HMA, GEO	Qualitative comparison between predicted metabolic consumption/productions and the metabolomic and lipidomic experimental measurements.
[180]	Warburg effect computer metabolic modeling	Study common and robust metabolic pathways supporting cancer cells (glycolysis, TCA cycle, pentose phosphate, glutaminolysis and oxidative phosphorylation). Propose metabolic targets for anticancer treatments by a constraint-based modeling on integrated data.	COBRA toolbox	GEO	Verified that the in silico kinetic growth curve exhibit a comparable behavior with the experimentally obtained from Hela CCLs.
[84]	Warburg effect computer metabolic modeling	GEM human metabolic network accounting stoichiometry and enzyme solvent capacity. Demonstrate that Warburg effect happens since the metabolic adaptation of cancer cells to increase biomass production rate.		BRENDA, SABIO-RK	Correlation between enzyme concentration predictions and expression of 1269 metabolic genes from 60 NCI CCLs. Validated against 1000 flux distributions of two models by ACHR sampling.
[181]	Warburg effect computer metabolic modeling	Expand metabolic efficiency notion by ATP production FBM constrained by glucose uptake and solvent capacities in the cell’s cytoplasm. Found that at low glucose uptake rates mitochondrial respiration is the most efficient pathway for ATP generation while when increasing glucose uptake rates a gradual switch to aerobic glycolysis achieves ATP highest rate since it is more efficient for the required solvent capacity.			Agreement between the experimentally determined fluxes and the model predictions.
[182]	Warburg effect computer metabolic modeling	Constraint-based modeling with E-Flux integrating 13 different cancer cell transcriptomics with Recon1 generic model. Found that metabolic changes distributions are similar in different cancer types, supporting that Warburg effect is a general metabolic adaptation.	E-Flux, GeWorkbench 2.4.0, COBRA	GEO	

Abbreviations: (C) carbon, (CCL) cancer cell line, (CRC) colorectal cancer, (CSGEM) cancer-specific GEM, (DIRAC) DIfferential RAnk Conservation, (GDC) Genomic Data Commons, (GEO) Gene Expression Omnibus, (GO) Gene Ontology, (HCC) Hepatocellular Carcinoma, (HMA) Human Metabolic Atlas, (HMD) Human Metabolome Database, (HPA) Human Protein Atlas, (HpaA) Human Protein Pathology Atlas, (N) nitrogen, (PC) prostate cancer, (RMGEM) reduced metabolic GEM.
